# Is It Worth It? Obesity Affects Snack Food Valuation Across the Menstrual Cycle

**DOI:** 10.3389/fnins.2022.800976

**Published:** 2022-02-16

**Authors:** Larissa S. Heuberger, Susanna Gobbi, Susanna C. Weber, Gwendolyn Graf, Philippe N. Tobler, Lori Asarian, Nori Geary, Mareike Roth, Brigitte Leeners

**Affiliations:** ^1^Swiss Federal Institute of Technology, Zurich, Switzerland; ^2^Zurich Center for Neuroeconomics, University of Zurich, Zurich, Switzerland; ^3^Department of Reproductive Endocrinology, University Hospital of Zurich, Zurich, Switzerland; ^4^Department of Medicine, University of Vermont, Burlington, VT, United States; ^5^Retired, Underhill, VT, United States; ^6^Department of Reproductive Endocrinology, University of Zurich, Zurich, Switzerland

**Keywords:** obesity, value-based decision making, food valuation, willingness to pay, ovarian hormones, menstrual cycle

## Abstract

**Background:**

The importance of menstrual cycle physiology in appetite and obesity is poorly understood. We investigated the effects of body mass index (BMI), menstrual cycle phase and sweet and salty taste on monetary valuation of snack foods.

**Methods:**

We recruited 72 women and after the application of in- and exclusion criteria 31 participants with healthy weight and 25 with obesity remained. The participants completed a willingness to pay (WTP) task to measure subjective value of 30 snack food items in the pre-ovulatory and mid-luteal cycle phases.

**Results:**

Generalized linear mixed model (GLMM) analysis revealed that BMI, cycle phase and snack taste interacted to influence WTP (−0.15 [−0.22, −0.03], *p* = 0.002). Hence, WTP was inversely related to BMI, but the strength of the relation depended on cycle phase and taste. The WTP of participants with healthy weight for salty taste changed across cycle phase but the WTP for sweet taste was not affected by cycle phase. Moreover, the cycle effect for the salty snacks ceased in participants with obesity.

**Conclusion:**

The inverse effect of BMI on WTP valuation of snack foods contrasts with the positive effect of BMI on pleasantness ratings for milkshakes by the same women that we previously reported. This indicates that the two measures reflect different aspects of food-related valuative processing in obesity. Furthermore, the WTP data suggest that the selection of salty snacks may differ from that of sweet snacks in the pre-ovulatory phase of the menstrual cycle for individuals of healthy weight. The cycle phase does not seem to affect food valuation of participants with obesity. These findings are relevant to understanding and treating obesity in women.

## Introduction

Obesity remains a pressing issue worldwide. In many countries, obesogenic environments are thought to contribute substantially to obesity’s high incidence. These environments include easily available energy-dense foods, which has brought about a relatively novel way of eating known as snacking (Duffey and Popkin, 2011; Mattes, 2018). The calorie intake from snacks add to the calorie intake from main meals, and therefore snacking is associated with a higher body mass index (BMI; kg/m^2^) (Mattes, 2018).

Nevertheless, despite the wide-ranging academic attention devoted to obesity, the roles of reproductive hormones in obesity in women have not often been taken into account. This is important because more women than men suffer from obesity worldwide (NCD Risk Factor Collaboration, 2016). Emotional eating, which is frequently associated with obesity, seems most frequent in the mid-luteal phase of the menstrual cycle and to be influenced by increased levels of both progesterone and β-estradiol (Klump et al., 2013). Indeed, many studies have shown increased subjective appetite and food intake in the mid-luteal compared to the mid- to late-follicular and periovulatory phases of the cycle (Buffenstein et al., 1995; Dye and Blundell, 1997; Brennan et al., 2009; Asarian and Geary, 2013; Gorczyca et al., 2016). It is unclear whether these changes are related to altered macronutrient selection. Some studies did not detect changes (Martini et al., 1994; Brennan et al., 2009), whereas others reported increased protein or carbohydrate intake in the mid-luteal phase (Bowen and Grunberg, 1990; Gorczyca et al., 2016). This change in intake could influence the nutrient metabolism in the gut affecting reward signals. In a recent study (de Araujo et al., 2020), the authors suggest that internal signals *via* the gut-brain-axis generate an unconscious food reward which might constitute the prime driver of overeating. This mechanism is part of a two-roads-to-food-reward model, where food reward is substantially influenced by the flavor-nutrient learning process happening in the gut (i.e., the low-road) and results not only from conscious reward signals based on food perception, taste and oral somatosensation (the high-road). Furthermore, a number of differences in taste perception have been linked to obesity and reproductive hormone status (Asarian and Geary, 2013; Hardikar et al., 2017). These studies indicate that better understanding of the effects of reproductive physiology and food type are important for continued progress in obesity research.

Ovarian hormones may affect food intake by changing the motivation to eat. For example, in ovariectomized rats, administration of β-estradiol resulted in decreased motivation for sucrose rewards (Richard et al., 2017). Such data suggest that estrogens and other ovarian hormones might modulate value-based decision making in relation to food. In addition, diet-induced obesity of female rats is associated with decreased dopamine signaling independent of the menstrual cycle in the nucleus accumbens, which is a fundamental brain region for reward processing (Geiger et al., 2009). We previously analyzed perceived pleasantness of milkshakes in relation to patterns of brain activity detected with fMRI in women with obesity or healthy weight (Gobbi et al., 2020). In that study there was an apparent effect of menstrual cycle phase on perceived pleasantness of sweet milkshakes, but this did not reach statistical significance (Gobbi et al., 2020). Here we expand on these findings studying in the same group of women another aspect of appetite, food valuation as an integration of hunger signals, past experience of the food’s flavor and its gastrointestinal and metabolomics effects. To this aim, we used the willingness to pay (WTP) paradigm. The WTP measures the motivation to eat using a bidding and auction method which was developed to quantify an option’s utility (Becker et al., 1964). In this study, we aimed to determine if women’s valuation of snack foods, first, varies between women with obesity and women with healthy weight, second, varies between the pre-ovulatory and mid-luteal phases of the menstrual cycle, and, third, whether these effects are similar for sweet and salty snack foods.

## Materials and Methods

All procedures were approved by the Ethics Commission of the Canton of Zurich, and the participants gave written informed consent. They were compensated for expenditures associated with study participation and received 500 Swiss Francs (CHF) for completing the study.

### Participants

The participants were recruited and screened for eligibility at the department of Reproductive Endocrinology (University Hospital Zurich). In the pre-screening phase, general, endocrinologic and mental health were assessed through a Clinical Assessment Questionnaire designed for the present study. Only individuals fulfilling our previously described (Gobbi et al., 2020) in- and exclusion criteria were recruited. Women with healthy weight (BMI 18.5–24.9 kg/m^2^) or with grade 1 or 2 obesity (BMI 30–39.9 kg/m^2^) and who reported that they were weight stable and not dieting were invited to participate. Eligible women were provided with the Three-Factor Eating Questionnaire (TFEQ) and the Eating Disorder Examination-Questionnaire (EDE-Q) to complete at home before the first test visit and to return by mail.

We recruited 72 physically and psychiatrically healthy women who were cycling normally and not using hormonal contraception. Four women withdrew from the study prior to completion, one was excluded due to insufficient German language skills, and two were excluded because of technical difficulties with the tasks. The participants were invited to attend two test visits, one in the pre-ovulatory phase and one in the mid-luteal phase of the menstrual cycle, with order randomized. Tests were timed in relation to menstrual cycle phases, calculated based on cycle information obtained from the participants (i.e., cycle length and days since the last menstruation) and confirmed against progesterone levels. For this paper, the pre-ovulatory phase targeted measurements from 16 ± 2 days prior to menstruation, i.e., shortly before ovulation, with low progesterone levels (3.0 ± 7.9 nmol/l). The mid-luteal phase targeted 7 ± 3 days prior to menstruation with high progesterone levels (21.4 ± 18.5 nmol/l). Six participants were excluded because their blood hormone levels were not in agreement with calculated cycle phases in either cycle phase. Another six participants for which only one cycle phase could be confirmed were retained, with their missing data accounted for in the statistical analyses. Three participants were excluded because their mean cycle length was not within the normal range (25–35 days).

### Test Procedure

Participants were advised not to drink or eat anything after 10pm on the evening prior to both test visits. The study compensation fee served as credit for the WTP task. In this task, participants bid money on a scale from 0 to 2.5 CHF in accordance with their desire to obtain different food items, which were pictures of 15 sweet and 15 salty common snack foods displaying a serving size of the product in front of its package on a black background. The snacks predominantly comprised chocolate bars, nut bars, cookies, gummy bears, crackers, olives, crisps, and salted nuts. To account for potential successive contrast influences among food items, this procedure was done twice for each food item in different order, resulting in 60 WTP bids. The participants bid on a continuous scale using a trackball, which they moved to the desired bid amount and then pressed to confirm their choice. The compensation method for the task was determined by an incentive-compatible auction mechanism (Becker et al., 1964). At the end of each session, one trial was selected at random and implemented. If the participant’s bid for the food item in that trial was greater than or equal to the auction price, they paid the auction price from their compensation fee and they received the snack to consume at the laboratory, before leaving. Otherwise, the participant did not obtain the food item and kept the entire compensation amount. The WTP task and several other tasks were completed whilst lying supine in a whole-body MRI scanner [Philips Medical Systems, Laboratory for Social and Neural Systems research (SNS Lab) in Zürich University Hospital, Zürich, Switzerland; more details regarding the scanning procedure and the other experiments are described elsewhere (Gobbi et al., 2020)]. In addition to the WTP task reported here, participants performed a task assessing food value measured by willingness to exert physical effort and a task related to experienced food value (milkshake sampling), which was previously reported (Gobbi et al., 2020). Trials of the different tasks were presented in random order. In addition, on each test day, participants performed the tasks in the MRI twice, once before and once after an *ad libitum* meal. The meal consisted of ham sandwiches and tap water, and the consumed amount was determined by comparing the weight of sandwiches before and after the meal. Weights were transformed to kcal and analyzed as *ad libitum* consumption level. Participants had 30 min to finish the meal and then returned to the scanner and repeated the three valuation tasks. Hence, the participant performed the tasks in two satiety states (fasted, fed) and two menstrual cycle phases (pre-ovulatory, mid-luteal), resulting in four sessions in total.

At different time points throughout the experiment, participants rated a number of subjective states. These included hunger, satiety, desire to eat, nausea, tiredness, feeling well, anxiety, discomfort, agitation, and dizziness. The participants provided these ratings using a generalized visual analog scale ranging from “not at all” to “as strong as possible” (Blundell et al., 2010). The participants answered by moving a trackball along the rating scale and clicking to indicate their response. For both WTP and subjective state ratings the scale direction was randomized across trials and sessions to disentangle brain activation from physical skills or habits related to moving the trackball.

### Statistical Analysis

Statistical analyses were performed using Microsoft Excel 2013 and R version 4.0.2 (R Core Team, 2013). Participants were weighed during both visits, which resulted in two different BMIs per participant (kg/m^2^). For the Spearman correlation analyses and the generalized linear mixed model (GLMM) analysis, BMI was treated as a continuous variable, whereas for group comparisons, we used the categorical variable weight status, i.e., healthy weight or obese.

**FIGURE 1:**
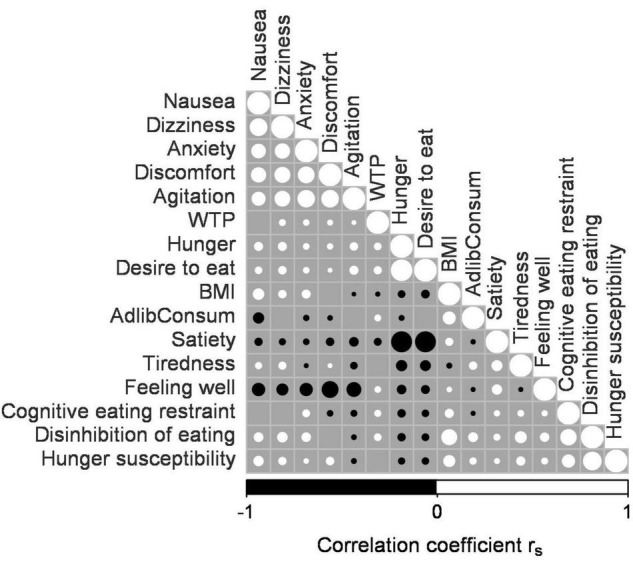
Correlation matrix of willingness to pay (WTP), *ad libitum* consumption level, BMI, Three-Factor Eating Questionnaire (TFEQ) scores, and subjective state ratings. This correlation matrix displays the correlation of WTP, BMI, *ad libitum* consumption level, TFEQ Cognitive eating restraint, TFEQ Disinhibition of eating, TFEQ Hunger susceptibility, nausea, dizziness, anxiety, discomfort, agitation, hunger, desire to eat, satiety, tiredness, and feeling well. See Results for further explanations. The diameter of the filled and open circles are proportional to the correlation magnitude (r_*s*_) where a large circle stands for a high r_*s*_. Open circles indicate positive correlation, whereas filled circles indicate negative correlations. No circle is depicted at intersections where there was no significant correlation (*p* > 0.05).

Willingness to pay bids without confirmation (no trackball press) were considered invalid, i.e., entered the analysis as missing values. In addition, we excluded items with WTP bids of zero in all four experimental sessions due to an apparent dislike of these items. Visual inspection of the histograms and the quantile-quantile plots indicated that the WTP bids and several other dependent variables were not normally distributed. Therefore, non-parametric statistical analyses were done for all analysis except the GLMM, which was based on a beta distribution. We computed Spearman correlation coefficients (r_*s*_) for WTP bids, BMI, *ad libitum* consumption levels, TFEQ scores, and the subjective state ratings from the time points around the WTP task, excluding missing values. A correlation matrix (Figure 1) was constructed using the R function corrplot (Wei et al., 2017). Magnitudes of the correlations are reported with the Spearman correlation coefficient r_*s*_.

We also contrasted the mean WTP bids, the EDE-Q and the TFEQ scores of the groups with healthy weight and obesity using Wilcoxon signed-rank tests for independent samples and we conducted group comparisons for WTP differences for sweet or salty food items using Wilcoxon signed-rank tests for paired samples. To calculate effect sizes r for the Wilcoxon tests we used the R function wilcox_effsize (Kassambara, 2020). We further tested for mean differences in WTP across cycle phases. To account for seven participants who completed two test visits in the same cycle phase and six participants for whom only one cycle phase was confirmed, we applied a method designed for paired data with missing values (Fong et al., 2018) using the R function pm.wilcox.test with the SR-MW method, which consists of a combination of signed-rank statistics for paired data and Mann-Whitney statistics for unpaired data (Fong et al., 2018). Because this test results in a *Z*-value, the effect sizes were calculated as the *Z*-statistic divided by the square root of the number of observations (Rosenthal, 1994). Visualizations of group comparisons were obtained using ggplot (Wickham, 2006). The Spearman correlations and group contrasts were considered exploratory, so we used a significance level of ɑ = 0.05.

The dependent variable for the GLMM analysis was the WTP. As these data followed an extreme values distribution determined by the monetary rating boundaries of 0 and 2.5 (in CHF), we used a beta regression model and a logit function as link (Cribari-Neto and Zeileis, 2010). The data were divided by 2.5 to normalize WTP values (WTP_*Norm*_) to between 0 and 1, as previously suggested (Smithson and Verkuilen, 2006), and transformed using the function WTP_*conv*_ = ([WTP_*Norm*_(n−1) + 0.5]/n) where n is the number of samples (Smithson and Verkuilen, 2006). We used the glmmTMB R package because it enables inclusion of random effects in the model (Brooks et al., 2017). We included random intercepts for participants and items as well as participant-specific random slopes for *Day* and *Satiety*. The model is defined as follows:


WTPij=(β+0u0j+u0k)+(β+1u1j)Dayij+(β+2u2j) Satietyij+βCPij3+βAdlibConsumij4+βBMIij5+β6Tasteij+βAdLibConsumij7BMIij+βCPij8AdLibConsumij+βCPij9BMIij+βBMIij10Tasteij+βCPij11Tasteij+βAdLibConsumij12Tasteij+βCPij13AdLibConsumijBMIij+βCPij14BMIijTasteij+βCPij15AdLibConsumijTasteij+eij

The index i represents the trial, j the participant and k the item. The continuous variables *BMI* and *ad libitum* consumption level (*AdlibConsum*) were *z*-scored at a group level and day of test visit (*Day*; Day 1 or Day 2), *Satiety* (Fasted or Fed), cycle phase (*CP*; Pre-ovulatory or Mid-luteal), and *Taste* (Sweet or Salty) were used as binary variables. Two- and three-way interactions were included. We calculated the *p*-values using the Wald statistic. To correct for the multiple tests using WTP bids as dependent variable (correlation, three Wilcoxon tests and the GLMM), we applied the Bonferroni procedure, resulting in a nominal threshold of 0.01 (= 0.05/5), and only effects for which *p*-value < 0.01 were considered significant. To display significant interaction effects we used the R functions ggpredict and ggplot (Wickham, 2006; Lüdecke, 2018).

## Results

### Subjects

We report the results for 56 women who fulfilled our selection criteria. Table 1 shows the participants’ demographic data. We assessed the cycle phases more conservatively than previously, resulting in fewer participants than in our previous paper (Gobbi et al., 2020). Forty-six (82%) women submitted complete questionnaires. These participants’ global EDE-Q scores were 1.4 ± 1.1, which closely matches community norm values (Fairburn et al., 2014). The EDE-Q scores Restraint, Eating concern, Shape concern and Weight concern were all significantly lower for participants with healthy weight than for those with obesity (median Restraint 0.4 and 1.2, *r* = 0.36, *p* = 0.016; Eating concern 0.2 and 0.8, *r* = 0.51, *p* < 0.001; Weight concern 0.1 and 3.0, *r* = 0.68, *p* < 0.001; and Shape concern 1.1 and 3.5, *r* = 0.65, *p* < 0.001). The TFEQ scores cognitive eating restraint and hunger susceptibility did not differ between participants with healthy weight and with obesity (*p* = 0.414 and *p* = 0.058) but disinhibition of eating was higher in the obese than in the healthy weight group (median 9.0 and 7.0, *r* = 0.40, *p* = 0.007).

**TABLE 1:** Demographics of the participants fulfilling the inclusion criteria.

	Mean ± SD	Range
Age (y)	25.5 ± 4.7	(18–40)
Healthy weight (*n* = 31)	26.0 ± 5.0	(19–40)
Obese (*n* = 25)	24.9 ± 4.4	(18–33)
BMI (kg/m^2^)	26.9 ± 5.4	(18.8–37.4)
Healthy weight (*n* = 31)	22.3 ± 2.1	(18.8–25.9)
Obese (*n* = 25)	32.3 ± 2.2	(29.0–37.4)
Cycle length (d)	29 ± 2	(25–35)
Healthy weight (*n* = 31)	28 ± 2	(25–32)
Obese (*n* = 25)	29 ± 2	(27–35)
EDE-Q: restraint	1.0 ± 1.0	(0–3.6)
Healthy weight (*n* = 27)	0.6 ± 0.8	(0–3.2)
Obese (*n* = 19)	1.4 ± 1.2	(0–3.6)
EDE-Q: eating concern	0.6 ± 0.8	(0–3.0)
Healthy weight (*n* = 27)	0.3 ± 0.4	(0–1.4)
Obese (*n* = 19)	1.1 ± 1.0	(0–3.0)
EDE-Q: shape concern	2.1 ± 1.5	(0–5.4)
Healthy weight (*n* = 27)	1.3 ± 0.8	(0–3.6)
Obese (*n* = 19)	3.3 ± 1.4	(0.8–5.4)
EDE-Q: weight concern	1.8 ± 1.5	(0–5.2)
Healthy weight (*n* = 27)	0.9 ± 0.7	(0–2.4)
Obese (*n* = 19)	3.0 ± 1.4	(0.4–5.2)
TFEQ: cognitive eating restraint	6.9 ± 4.0	(1–15)
Healthy weight (*n* = 27)	6.5 ± 4.4	(1–14)
Obese (*n* = 19)	7.5 ± 3.5	(2–15)
TFEQ: disinhibition of eating	7.1 ± 3.1	(1–15)
Healthy weight (*n* = 27)	5.9 ± 3.2	(1–11)
Obese (*n* = 19)	8.7 ± 3.5	(2–15)
TFEQ: hunger susceptibility	5.3 ± 3.6	(0–13)
Healthy weight (*n* = 27)	4.6 ± 2.9	(0–11)
Obese (*n* = 19)	6.3 ± 3.1	(1–13)

*Data are mean ± SD and range for all 56 participants except as noted. BMI [weight (kg)/height^2^ (m^2^)] are the data collected on the two test days. The women with healthy weight include three women whose BMI increased from below 25 at screening to between 25 and 26 during the tests, and the women with obesity include three women whose BMI decreased from above 30 at screening to between 29 and 30 during the tests.*

### Spearman Correlations

Figure 1 summarizes the Spearman correlational data. The WTP bids correlated very weakly positively with desire to eat, hunger, feeling well, dizziness, anxiety, agitation and discomfort (*r*_*s*_ ≤ 0.1, *p* ≤ 0.011) and negatively with satiety rating (*r*_*s*_ = −0.09, *p* < 0.001) and BMI (*r*_*s*_ = −0.03, *p* = 0.002). Analysis of the TFEQ scores revealed a weak but significant correlation of BMI with cognitive eating restraint and hunger susceptibility (*r*_*s*_ = 0.13, *p* < 0.001 and *r*_*s*_ = 0.25, *p* < 0.001) and a moderate correlation of BMI with disinhibition of eating (*r*_*s*_ = 0.46, *p* < 0.001). We also found very weak correlations of WTP with cognitive restraint and with disinhibition of eating (*r*_*s*_ ≤ 0.1, *p* < 0.001; Figure 1). Hence, the Spearman correlations of WTP bids with the subjective state ratings, TFEQ scores and *ad libitum* consumption level failed to reveal any strong relationships.

### Group Comparisons

**FIGURE 2:**
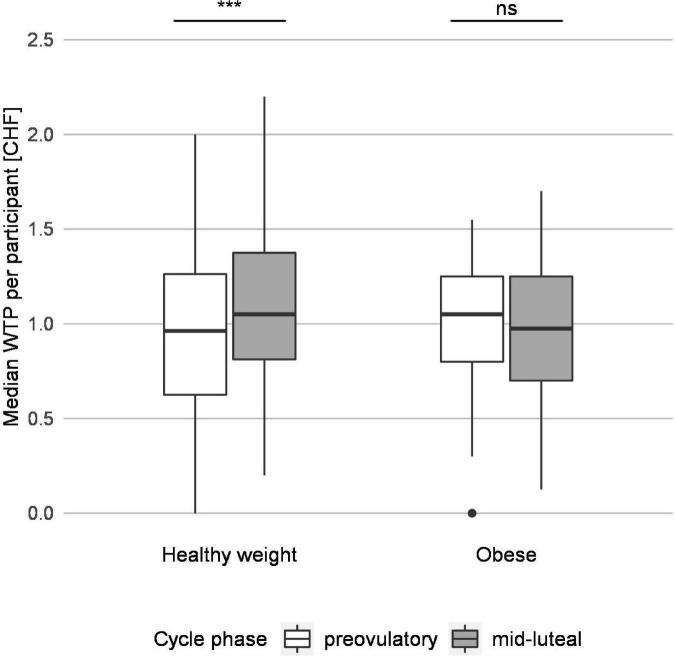
Group testing reveals that pre-ovulatory and mid-luteal willingness to pay (WTP) differs for participants with healthy weight but not for participants with obesity. The median WTP per participant for participants with normal weight and with obesity are depicted. The white and the gray boxes represent the median WTP per participant in the pre-ovulatory and mid-luteal cycle phase, respectively, and indicate the range of the data from the first to the third quartile. The horizontal line represents the median, and the whiskers reach to the minimal and maximal values not considered outliers; one outlier is represented by a dot. Group comparisons revealed a significant difference in WTP between pre-ovulatory and mid-luteal cycle phase for participants with healthy weight but not for participants with obesity. ***p < 0.001, ns, not significant.

The WTP bids (in CHF) of participants with healthy weight were significantly reduced in the pre-ovulatory compared to the mid-luteal phase (median 0.95 and 1.10, *r* = −0.14, *p* < 0.001), whereas those of women with obesity did not change significantly across cycle phases (*p* = 0.334; Figure 2). Furthermore, group comparisons within cycle phase showed that the pre-ovulatory WTP bids were higher in participants with obesity than in participants with healthy weight (median 1.00 and 0.95, *r* = 0.05, *p* = 0.012) whereas the mid-luteal WTP bids were lower in participants with obesity than those with healthy weight (median 1.00 and 1.10, *r* = 0.07, *p* < 0.001). Thus, obesity eliminated the effect of the menstrual cycle on WTP for snack foods that was evident in women with healthy weight (Figure 2).

On average, the WTP bids for sweet snacks were higher than the ones for salty snacks (median 1.08 and 0.98, *r* = 0.13, *p* < 0.001). This difference arose in both cycle phases (pre-ovulatory *r* = 0.14, *p* = 0.002 and mid-luteal *r* = 0.12, *p* = 0.010) and weight groups (healthy weight *r* = 0.12, *p* = 0.010 and obese *r* = 0.15, *p* = 0.002). Hence, WTP differed depending on the taste of the snack foods presented, i.e., sweet or salty.

### Generalized Linear Mixed Model

**FIGURE 3:**
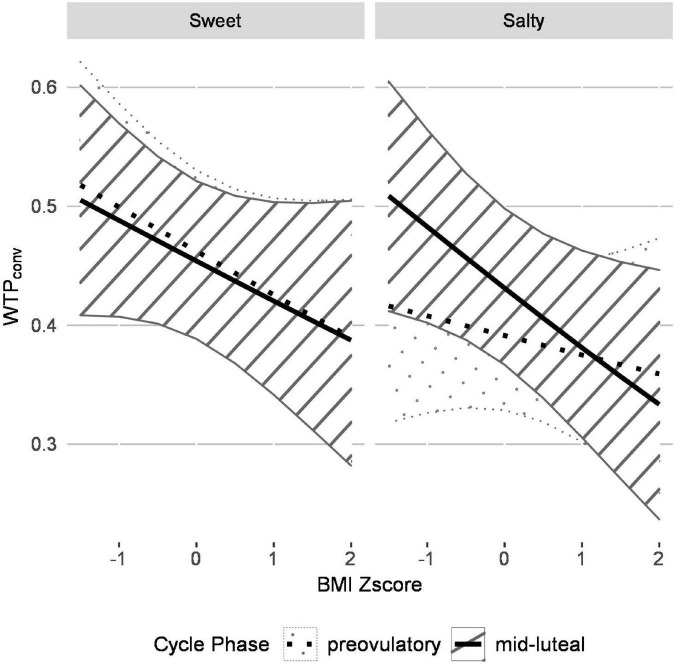
The generalized linear mixed model (GLMM) reveals that the relationship between WTP_*conv*_ is inversely related to BMI for both sweet and salty snacks and menstrual cycling affects WTP_*conv*_ for salty, but not for sweet, snacks. The plot visualizes how cycle phase, taste and BMI interact to predict WTP_*conv*_ according to the GLMM. WTP_*conv*_ is inversely related to BMI and the strength of this relationship depends on cycle phase and taste. For sweet food items, average pre-ovulatory and mid-luteal WTP_*conv*_ both decrease with increasing BMI and the GLMM only predicted a slight difference between cycle phases. For salty food items, higher average WTP bids are predicted in the mid-luteal compared to the pre-ovulatory cycle phase for participants with low BMI whereas this effect diminishes with increasing BMI. Control analyses verified that the overall effect was reflected in the data of the individual groups.

The GLMM outcomes are summarized in Table 2. The satiety state significantly affected WTP_*conv*_ (*p*_*corrected*_ < 0.01). Specifically, average WTP_*conv*_ was higher in fasted compared to fed satiety state (*Z* = −5.13, *p* < 0.001). The predictor snack type (“taste”) showed a trend-level effect but did not survive Bonferroni correction. There were two significant interactions predicting WTP_*conv*_ bids (*p*_*corrected*_ < 0.01). The first interaction was between snack type and cycle phase (*Z* = 4.27, *p* < 0.001), the second one between snack type, cycle phase and BMI (*Z* = −3.07, *p* = 0.002). As shown in Figure 3, WTP_*conv*_ was inversely related to BMI but the strength of the relation depended on cycle phase and snack type. That is, for both snack types in the mid-luteal phase and for sweet snacks in the pre-ovulatory phase, mean WTP_*conv*_ decreased similarly from ∼0.50 in participants with the lowest BMI to ∼0.35 in participants with the highest BMI. In contrast, WTP_*conv*_ for salty snacks in the pre-ovulatory phase was less, ∼0.41, in participants with the lowest BMI, and decreased less steeply, to ∼0.37 for participants with the highest BMI. Furthermore, WTP bids for salty foods were generally higher in women with lower BMI than with high BMI, especially during the mid-luteal phase. In contrast, for sweet snacks, WTP bids were also generally higher in women with lower BMI, but were not affected by menstrual cycle phase.

**TABLE 2:** The generalized linear mixed model (GLMM) reveals a main effect of satiety state, an interaction of cycle phase with snack taste, and a three-way interaction of cycle phase with snack taste and BMI to significantly predict WTP_*conv*_.

	Estimate	SE	*Z*-statistic	*P*-value
(Intercept)	−0.15 [−0.34, 0.09]	0.14	–1.12	0.264
Mid-luteal	−0.04 [−0.21, 0.15]	0.09	–0.40	0.687
AdlibConsum	0.08 [−0.11, 0.25]	0.09	0.86	0.391
BMI	−0.15 [−0.33, 0.06]	0.10	–1.45	0.146
Salty	−0.29 [−0.36, −0.23]	0.12	–2.37	0.018
Fed	−0.24 [−0.32, −0.14]	0.05	–5.13	< 0.001*
Day 2	−0.03 [−0.18, 0.13]	0.08	–0.36	0.717
AdlibConsum × BMI	0.05 [−0.11, 0.22]	0.08	0.65	0.515
Mid-luteal × AdlibConsum	0.10 [−0.09, 0.30]	0.10	1.00	0.319
Mid-luteal × BMI	0.01 [−0.18, 0.18]	0.09	0.15	0.878
BMI × Salty	0.08 [−0.01, 0.13]	0.04	2.21	0.027
Mid-luteal × Salty	0.20 [0.09, 0.28]	0.05	4.27	< 0.001*
AdlibConsum × Salty	−0.04 [−0.11, 0.04]	0.04	–1.12	0.263
Mid-luteal × AdlibConsum × BMI	−0.07 [−0.25, 0.10]	0.09	–0.73	0.466
Mid-luteal × BMI × Salty	−0.15 [−0.22, −0.03]	0.05	–3.07	0.002*
Mid-luteal × AdlibConsum × Salty	−0.05 [−0.16, 0.04]	0.05	–1.03	0.303

Number of observations:	11,022			
BIC:	−14655.2			

This table illustrates the GLMM results aiming to explain WTP_conv_ by the different predictors CP (Pre-ovulatory or Mid-luteal), AdlibConsum (Z score), Taste (Sweet or Salty), BMI (Z score), Satiety (Fasted or Fed), Day (Day 1 or Day 2), and interaction effects among predictor variables, according to Eq. 1. The confidence intervals were computed for the fixed terms considering the random effect for participants but not items because it was not possible to include both random effects due to algorithmic limitations of the package used in R. The p-values were obtained using the Wald Z-statistic. Bonferroni-corrected significant effects were found for Satiety (Fed), the interaction of CP and Taste (Mid-luteal × Salty) as well as the interaction of BMI, CP, and Taste (BMI × Mid-luteal × Salty). Data are mean regression coefficients [95% CI] and all continuous regressors were z-scored before entering the model. P-values are uncorrected; *Significant following Bonferroni-correction, p_corrected_ < 0.01.

## Discussion

### Food Valuation Depends on Obesity, Cycle Phase, and Snack Taste

In this study we used a WTP method to measure the subjective value of snack foods in women with healthy weight or obesity. We report an inverse relationship between WTP and BMI, which depends on snack taste and cycle phase. The decreasing WTP with increasing BMI is opposite to what we found in a milkshake tasting task, where the same participants’ pleasantness ratings increased with BMI (Gobbi et al., 2020). This indicates that the different tasks link to different parts of the reward and valuation systems. Specifically, rating milkshake pleasantness is a more sensory or consummatory process, whereas WTP is a more anticipatory process. Moreover, compared to typical anticipatory ratings, such as expected satiety (Brunstrom, 2014), the WTP task is a more complex decision-making and value-estimation task. Indeed, during the milkshake task our fMRI data indicated that predominantly hedonic and homeostatic brain circuits were activated (Gobbi et al., 2020) whereas WTP has been reported to be associated with activity in different regions of the prefrontal cortex (Plassmann et al., 2007; Tang et al., 2014). This might reflect involvement of cognition and value computation in the WTP task, which is in line with the prefrontal cortex being involved in cognitive control of goal-directed behavior (Miller and Cohen, 2001). Relatedly, cognitive restraint of eating may have influenced WTP bidding of participants with obesity. High cognitive restraint is positively associated with BMI [e.g., Banna et al. (2018) and Adams et al. (2019)] and has been reported to dampen cyclic eating changes (Asarian and Geary, 2013). We measured a small positive correlation of both WTP and BMI with the TFEQ score cognitive eating restraint and the EDE-Q scores Restraint and Eating concern were significantly increased in women with obesity. Hence, WTP of participants with obesity might be lower in part due to cognitive restraint.

### Food Valuation Differs by Cycle Phase for Salty Snacks

Our findings reveal that valuation appears to be higher for sweet than for salty snack foods, especially for participants with obesity and in the pre-ovulatory cycle phase. In contrast, mid-luteal WTP bids of participants with healthy weight did not differ by taste. Hence, while the WTP for salty snacks increased from the pre-ovulatory to mid-luteal cycle phase, the WTP for sweet snacks did not depend on cycle phase. This absence of cycle effect for sweet food parallels the findings on pleasantness of milkshakes (Gobbi et al., 2020). The mid-luteal increase in women’s value for salty food is in accordance with an apparent stronger dislike of unsalted popcorn in the luteal phase compared to the follicular phase (Frye and Demolar, 1994). However, neither intake of nor preference ratings for salty food changed between menstrual cycle phases (Bowen and Grunberg, 1990). Thus, further research is required to assess the relationship between the valuation change and actual intake of salty foods in snack and non-snack contexts. We conclude, first, that women’s food valuation differs by taste and, second, that valuation for salty food but not for sweet food differs by cycle phase.

### Possible Mechanisms

Previous neuroimaging studies demonstrated correlations between WTP and activity of different brain regions (Plassmann et al., 2007; Tang et al., 2014). Some of these are associated with subjective values, reinforcing the idea that the WTP approach is a valid measure of subjective value (Bartra et al., 2013). We found that WTP is lower in the pre-ovulatory than in the mid-luteal phase for participants with healthy weight. In line with this, another study found that the appeal of food images was lower in the second week of the menstrual cycle than in the last week (Frank et al., 2010). Such changes across the menstrual cycle have also been reported for brain activation in response to food images and uncertain monetary rewards (Dreher et al., 2007; Frank et al., 2010). Furthermore, rat studies have revealed that estrogens enhance striatal dopamine signaling (Becker and Cha, 1989; Becker, 1999; Yoest et al., 2014). Although neither estrogens nor progestins are sufficient to explain increased energy intakes in the mid-luteal cycle phase in women or other anthropoid primates (Asarian and Geary, 2013), increases in the two hormones have been associated with increases in emotional eating during the mid-luteal phase (Klump et al., 2013) and progesterone and β-estradiol synergized to induce striatal dopamine release in rats (Yoest et al., 2018).

As described above, WTP bids decreased with increasing BMI, but changed across the menstrual cycle only in participants with healthy weight bidding for salty snacks. At least the former effect may be related to dopamine signaling. The reward behavior in the group with obesity, which did not depend on the menstrual cycle, supports results of experiments with female rats which showed that an obesity-inducing regimen decreased mesolimbic dopamine transmission (Geiger et al., 2009). Furthermore, reduced activation of reward-associated brain regions in individuals with obesity has been shown in human trials (Carnell et al., 2012), and striatal dopamine D_2_ receptor expression was lower in individuals with severe obesity compared to humans with healthy weight (Wang et al., 2001). Relatedly, long term intake of energy-rich food (Burger and Stice, 2011) and high saturated-fat diets (Kleinridders and Pothos, 2019) decrease dopamine signaling in reward-associated brain areas. Thus, chronic downregulation of dopamine in certain brain regions of individuals with obesity may interfere with reward processing, which would otherwise depend on menstrual cycle. Further research should address how dopamine signaling affects valuation of different foods across the menstrual cycle and in women with different BMI.

### Subjective State Ratings and Satiety State

The GLMM revealed a significant main effect of satiety state (fed or fasted) as a predictor for WTP_*conv*_. This supports theoretical approaches suggesting that value-based decision making depends on hunger (Niv et al., 2006; Rangel et al., 2008) and that the food evaluation process is influenced by the gut-brain-axis (de Araujo et al., 2020). In apparent contrast, our correlational analyses suggested that WTP bids were not substantially related to subjective states. In view of the interactive effect of BMI, cycle phase and snack taste revealed by the GLMM, however, it is not surprising that simple correlational analyses failed. The lower power of non-parametric correlations may have also contributed. It is also possible that subjective ratings are weaker measures of food valuation than our behavioral and fMRI measures. For example, the weak correlation of satiety ratings and WTP contrasts with the clear main effect of satiety state as a predictor for WTP_*conv*_. Additionally, our fMRI analysis revealed increased striatal and prefrontal activity in participants with obesity despite their ratings of high satiety and low desire to eat (Gobbi et al., 2020).

### Strengths and Limitations

We used BMI as a metric to discriminate between people with and without obesity rather than other methods like dual energy x-ray absorptiometry (DXA) because it remains a simple and accurate measure for obesity on a population level (The World Health Organization [WHO], 2022). However, the association between BMI and body fat content is limited because BMI does not consider age, physical activity level, and sex (The World Health Organization [WHO], 2022). Still, in our study, we controlled for the variables age and sex as only adult, premenopausal women were included. Moreover, we asked the participants to fill out the International Physical Activity Questionnaire (IPAQ) to determine their physical activity (Craig et al., 2013) because it has been shown that BMI can be especially inaccurate for individuals with a high muscle mass, e.g., athletes. Indeed, the literature suggests that athletes’ food choices are influenced not only by the commonly found factors taste, convenience, and nutrition (Birkenhead and Slater, 2015). Sport requires a different type of energy supply and athletes choose food in order to optimize their performance (Parraga, 1990; Eertmans et al., 2005; Birkenhead and Slater, 2015). Likewise, weight-conscious individuals usually prioritize low-energy foods that are helpful for their diet and body composition compared to more palatable choices (Birkenhead and Slater, 2015). Thus, we could expect sports involvement to enhance inhibitory control and dietary self-control (Wills et al., 2007; Lowe et al., 2016) and consequentially to decrease the positive evaluation of food snacks in the WTP task implemented in the study. The IPAQ allowed us to control for differences in the level of physical activity between people with obesity and with healthy weight; on average between groups, we did not find any. Therefore, we can exclude high BMI due to increased muscle mass. Furthermore, the difference of body weight in an average Swiss woman (1.64 m tall; Eglitis-media, 2022) between a BMI of 22.3 and 32.3 (mean BMI of the two groups) corresponds to 26.9 kg, which is unlikely to be due to differences in muscle mass alone. Hence, in view of these considerable differences of BMI between groups and the control for confounding factors such as age, sex, and physical activity, we considered BMI as a sufficient measure to differentiate participants with healthy weight from those with obesity. In general, our inclusion criteria were rather strict in order to increase the likelihood of detecting causal neuroendocrine effects. Although we invited only participants with a BMI greater than 30 or below 25, some participants changed body weight enough to bring their BMI outside the inclusion criteria. This was unlikely to be of high importance for the majority of our analysis in which BMI was used as a continuous variable. Participants were informed that the study was about steroid hormones and asked to maintain menstrual cycle records; this might have promoted response biases related to their beliefs about menstrual cycle and appetite. Timing may have affected some outcomes because food preferences are lower in the morning, when our experiment took place, than later in the day (Reichenberger et al., 2018). Lastly, other factors, such as prices, social information, habituation and labels, might have influenced WTP ratings in our test situation (List and Shogren, 1999; Niv et al., 2006; Rangel et al., 2008; Suzuki et al., 2017; Motoki and Suzuki, 2020). To minimize habituation bias, we organized the study sessions in random order with respect to cycle phase. Furthermore, the participants were provided with all instructions prior to the test sessions and they conducted the WTP task alone in the scanner.

## Conclusion

This study aimed to measure food valuation and to investigate whether it changes with BMI, cycle phase, and taste. Our major findings were that WTP changes across the menstrual cycle for participants with healthy weight and for salty food items. This indicates that obesity research should focus more on the influences of, first, types of foods and eating occasions and, second, reproductive hormones on reward processing, in particular dopamine signaling and anticipatory food valuation. Furthermore, the reduction in the effect of menstrual cycle changes on food valuation in women with obesity is a novel finding which, if reproduced by future studies, may be critical for future obesity research.

## Data Availability Statement

The datasets generated and/or analyzed during the current study are available from the corresponding author on reasonable request. Requests to access the datasets should be directed to BL, brigitte.leeners@usz.ch.

## Ethics Statement

The studies involving human participants were reviewed and approved by Ethics Commission of the Canton of Zurich. The patients/participants provided their written informed consent to participate in this study.

## Author Contributions

LH planned and performed the data analysis and wrote the first draft of the manuscript. SG collected and curated the data, planned and supported the data analysis. SW programmed the task and collected data. GG, MR, and BL realized recruitment and selected the participants. BL, LA, NG, PT, MR, and SW designed the study. All authors contributed to the manuscript and approved the final version.

## Conflict of Interest

The authors declare that the research was conducted in the absence of any commercial or financial relationships that could be construed as a potential conflict of interest.

## Publisher’s Note

All claims expressed in this article are solely those of the authors and do not necessarily represent those of their affiliated organizations, or those of the publisher, the editors and the reviewers. Any product that may be evaluated in this article, or claim that may be made by its manufacturer, is not guaranteed or endorsed by the publisher.
